# Health Distress and Challenges in Patients With Myeloproliferative Neoplasms During Follow‐Up Consultations at the Outpatient Clinic: A Phenomenological Study

**DOI:** 10.1002/cnr2.70373

**Published:** 2025-11-06

**Authors:** Yating Liu, Jinying Zhao, Qianqian Zhang, Qingyan Gao, Yayun Cao, Jia He, Lihua Han, Yandi Wang, Qian Yang, Wenjun Xie

**Affiliations:** ^1^ State Key Laboratory of Experimental Hematology, National Clinical Research, Center for Blood Diseases, Haihe Laboratory of Cell Ecosystem, Institute of Hematology & Blood Diseases Hospital Chinese Academy of Medical Sciences & Peking Union Medical College Tianjin China; ^2^ Tianjin Institutes of Health Science Tianjin China

**Keywords:** experience, health distress, MPN, myeloproliferative neoplasms, outpatient clinic, qualitative research

## Abstract

**Background:**

Myeloproliferative neoplasms (MPN) present significant management challenges as their constitutional symptoms greatly affect patients' quality of life. While current literature addresses hematological aspects, the full symptom burden and post‐clinic challenges for adult patients, especially in outpatient settings, remain unclear.

**Aims:**

To systematically identify and characterize the health distress and challenges experienced by patients with MPN in mainland China.

**Methods and Results:**

This qualitative study employed semi‐structured interviews with adults diagnosed with MPN (*n* = 26) to explore their experiences in the outpatient clinic. Colaizzi's descriptive framework guided transcript analysis. Four themes and 12 subthemes were identified: (1) living with the illness, (2) the Sword of Damocles, (3) role conflict, and (4) systemic barriers. These themes informed the development of a trajectory describing the physical and psychological experiences of MPN patients after outpatient visits.

**Conclusion:**

Chinese MPN patients encounter significant health challenges, including persistent symptoms, psychological distress, social limitations, and inadequate self‐management. A comprehensive understanding of their longitudinal experiences is essential for designing effective interventions. Additional research should focus on integrating biopsychosocial management into routine follow‐up care.

## 1 Introduction

Myeloproliferative neoplasms (MPN) are clonal hematopoietic stem cell disorders. The “classic” Philadelphia chromosome‐negative (Ph‐) MPN, which includes polycythemia vera (PV), essential thrombocythemia (ET), and primary myelofibrosis (PMF), share similar phenotypes [1]. Patients with MPN experience ongoing symptom burdens that affect quality of life. These constitutional symptoms include fatigue, itching, night sweats, weight loss, fever, and bone pain [2]. All MPN subtypes can progress to acute leukemia, reducing survival once the disease enters the accelerated phase. PMF patients experience the most frequent transformations, with a 10‐year rate ranging from 5% to 30%. ET and PV have a 10‐year transformation rate ranging from 2.3% to 3.8% [3, 4]. This progression makes disease management challenging during stable outpatient follow‐up.

In recent years, clinical teams have utilized the MPNs symptom assessment form total symptom score (MPN‐SAF, also MPN‐10) to assess symptom burden. This tool offers a quantitative perspective on patient‐reported outcomes [5]. In China, studies with these tools show that patients with myelofibrosis (MF) have the highest symptom burden, followed by PV and ET [6, 7]. Shi et al.'s [8] research found that higher MPN‐10 scores were significantly associated with poorer quality of life and work productivity in patients with MPN. Additionally, Guo et al. [9] reported a positive correlation between mutation burden and white blood cell counts in MPN patients. These data quantify symptom burden and genomic profile, but they give little insight into why such burdens exist or the subjective experiences of Chinese patients.

Qualitative studies from other countries provide important insights into the experiences of MPN patients. In the Philippines, research showed that patients experience varied, overlapping, and dynamic symptoms. These symptoms affect productivity both at home and at work [10]. Rossau et al. [11] stressed the importance of psychosocial support and patient education. These factors help patients balance limited energy and set priorities for daily activities. Bradford et al. [12] highlighted that healthcare providers often neglect fatigue management, leaving patients with little education or support.

Outpatient follow‐up for MPN is common in China, but research on patient health issues during this phase is limited. To address this gap and enhance quality of life in China, more studies are needed to develop patient‐centered, evidence‐based guidelines. Accordingly, this study conducted in‐depth interviews with MPN patients during outpatient follow‐up to clarify their experiences, distress, management needs, and life challenges.

## 2 Methods

### 2.1 Design

A qualitative design using descriptive phenomenological analysis was applied [13]. According to Finlay [14], Husserl's descriptive phenomenology holds that “information and insight do not come from large amounts of data, but emerge from an intense study of experiences.” This approach was chosen to capture participants' lived experiences in the Chinese cultural context. The study followed the consolidated criteria for reporting qualitative research checklist (COREQ) [15].

### 2.2 Participants and Recruitment

From October 2023 to March 2024, the study was conducted at the outpatient clinic of the Myelodysplastic Syndrome and MPNs (MDS&MPN) Treatment Center, part of the Institute of Hematology & Blood Diseases Hospital, Chinese Academy of Medical Sciences (IHCAMS). The head nurse (JYZ) explained the study to the staff. A registered nurse (QQZ) led recruitment with support from an attending physician (QYG). Purposeful and maximum variation sampling ensured diverse, detailed participants across MPN subtypes, ages, and genders. Recruitment stopped when no new themes emerged [16].

The following inclusion criteria were adopted: (1) aged 18 years or older, (2) meeting the WHO classification and diagnostic criteria for MPN (2016 Edition) [17] and having been diagnosed for at least 6 months, (3) receiving outpatient follow‐up routinely for the past 6 months, and (4) having good communication and expression skills. Participants were excluded if they had a psychiatric illness, cognitive impairment, or language problems.

## 3 Data Collection and Analysis

### 3.1 Data Collection

Twenty‐eight patients were approached, and 26 consented to participate (response rate 93%). Two declined due to time constraints (*n* = 1) or unwillingness to discuss the topic (*n* = 1). Semi‐structured interviews were conducted in a private room located near the outpatient clinic. The first author, a trained hemato‐oncology nursing researcher, systematically observed and recorded both verbal and non‐verbal behaviors. All interviews were audio‐recorded using a voice recorder (iFLY TEK H1 Pro).

The initial interview guide was based on literature review [18] and study objectives and was refined after pilot interviews. The interview guide included six questions: (1) What symptoms and feelings concern you most now? (2) How do these symptoms or disease affect your daily life, activities, or work? (3) During outpatient follow‐up, what difficulties did you experience and how did you resolve them? (4) What important health distress or challenges are ignored by medical staff? (5) How do you feel about your relationships with others after your illness? and (6) Is there anything else you want to share about your experiences?

### 3.2 Data Analysis

Two researchers (YTL and JYZ) conducted the analysis collaboratively using Colzizzi's seven‐step method [19]. The process was supported by QSR International's NVivo 12 Pro qualitative data analysis software and involved the following: (1) the researchers immersed themselves in the data through repeated reading of transcripts; (2) significant statements related to MPN care were identified; (3) meaningful units within the text were derived through discussion; (4) all meaningful units were grouped into theme clusters; (5) each theme prototype was outlined and explained; (6) subthemes featuring common events were organized into overarching themes; and (7) findings were returned to participants to validate the authenticity.

### 3.3 Rigor

Multiple strategies ensured study rigor, addressing credibility, transferability, dependability, and confirmability [20]. The interviewer maintained field notes during data collection and analysis. Two researchers independently coded transcripts to minimize bias, with themes validated through team consensus. An experienced hematologist physician reviewed the analysis. Methodological records were maintained to support transferability.

### 3.4 Ethical Consideration

The study was approved by the Ethics Committee of the Institute of Hematology & Blood Diseases Hospital, Chinese Academy of Medical Sciences (No. FRFCU2023042‐EC‐2, Date: 2023/10/13). Prior to each interview, participants received a detailed study explanation with an assurance of data confidentiality and then provided written informed consent in accordance with the Declaration of Helsinki.

## 4 Results

### 4.1 Participant Characteristics

Twenty‐six participants were men (*n* = 14) and women (*n* = 12), aged 32–71 years old (mean 53.27, SD 11.21) with a diagnosis of PV (*n* = 11), PMF (*n* = 9), and ET (*n* = 6). The range of time since diagnosis was between 1 and 25 years. Physical examination findings, laboratory parameters, and MPN‐10 scores were obtained from participants during interviews within the preceding week. Table 1 summarizes the socio‐demographic and clinical information in detail. The assessment of MPN‐10 revealed that fatigue was the most prevalent constitutional symptom across all MPN subtypes (Table 2). Treatment regimens comprised aspirin, hydroxyurea, and/or interferon for most PV (92%) and ET (90%) patients, whereas 85% of PMF patients received diverse agents (e.g., interferon, hydroxyurea, thalidomide).

**TABLE 1:** Demographic and clinical data of the participants in MPN (*n* = 26).

Variables	PV (*n* = 11)	ET (*n* = 6)	PMF (*n* = 9)
Sex, *n* (%)			
Female	3 (27.3)	4 (66.7)	5 (55.6)
Male	8 (72.7)	2 (33.3)	4 (44.4)
Age (years), *n* (%)			
< 45	3 (27.3)	3 (50.0)	2 (22.2)
46–64	7 (63.6)	2 (33.3)	4 (44.5)
≥ 65	1 (9.1)	1 (16.7)	3 (33.3)
Marital status, *n* (%)			
Married	10 (90.9)	5 (83.3)	7 (77.8)
Unmarried	1 (9.1)	1 (16.7)	2 (22.2)
Education^a^, *n* (%)			
Primary school	3 (27.3)	0 (0)	3 (33.3)
Middle school	2 (18.2)	1 (16.7)	1 (11.1)
High school	0 (0)	3 (50.0)	2 (22.2)
College or higher	6 (54.4)	2 (33.3)	3 (33.3)
Employment status, *n* (%)			
Working	9 (81.8)	4 (66.7)	7 (77.8)
Not working	0 (0)	1 (16.7)	0 (0)
Retired	2 (18.2)	1 (16.7)	2 (22.2)
Comorbidity, *n* (%)			
Thrombotic events	4 (36.4)	2 (33.3)	3 (33.3)
Splenomegaly	2 (18.2)	1 (16.7)	5 (55.6)
Disease course (years), median (IQR)	4 (3.5, 7.5)	4 (3.3, 4.8)	2 (1.5, 3.0)
Hemoglobin (g/L), median (IQR)	175 (133, 208)	128 (110, 153)	97 (63, 116)
Platelets (10^9^/L), median (IQR)	217 (61, 457)	531 (146, 1270)	154 (21, 836)
White blood cells (10^9^/L), median (IQR)	6.6 (1.8, 20.3)	7.1 (4.7, 21.5)	13.5 (4.2, 63.7)
MPN‐10, mean (SD)	15.76 (11.43)	5.63 (3.76)	20.15 (13.22)

Abbreviation: MPN‐10, Myeloproliferative Neoplasms Symptom Assessment Form total symptom score (MPN‐SAF, also MPN‐10).

^a^ Primary school: Age 6–12, Middle school: Age 13–15, High school: Age 16–18, College and above: 3‐year college education or higher.

**TABLE 2:** Assessment of symptom burden based on the MPN‐10 (*n*, %).

Items	PV (*n* = 11)	ET (*n* = 6)	PMF (*n* = 9)
Fatigue (exhaustion)	8 (72.7)	3 (50.0)	8 (88.9)
Early satiety	2 (18.2)	1 (16.7)	7 (77.8)
Abdominal pain	2 (18.2)	0 (0)	6 (66.7)
Inactivity	5 (45.5)	2 (33.3)	6 (66.7)
Concentration problems	1 (9.1)	0 (0)	6 (66.7)
Night sweats	3 (27.3)	2 (33.3)	5 (55.6)
Itching	3 (27.3)	2 (33.3)	4 (44.4)
Bone pain	1 (9.1)	1 (16.7)	3 (33.3)
Fever	3 (27.3)	1 (16.7)	2 (22.2)
Weight loss	6 (54.5)	1 (16.7)	5 (55.6)

Abbreviation: MPN‐10, Myeloproliferative Neoplasms Symptom Assessment Form total symptom score (MPN‐SAF, also MPN‐10).

### 4.2 Findings of Transcripts

Each interview ranged from 30:08 to 47:15 (min:s) in duration, with an average length of 38:10 (min:s). Analysis yielded four overarching themes and 12 subthemes, capturing 119 codes, which reflected the experiences of patients during outpatient follow‐up (Table 3). The four themes included (1) living with the illness, (2) the Sword of Damocles, (3) role conflict, and (4) systemic barriers.

**TABLE 3:** Overview of themes and subthemes.

Themes	Subthemes
Theme 1: Living with the illness	Symptom burden
	Side effects
	Body image change
Theme 2: The sword of Damocles	Uncertainty of the future
	Fear of disease progression
	Cognitive avoidance
Theme 3: Role conflict	Restricted family role
	Resistance to returning to work
	Reluctant to disclose the condition
Theme 4: Systemic barriers	Lack of professional support
	The Information paradox
	Lack of empowerment

### 4.3 Theme 1: Living With the Illness

MPN patients perceived their condition as a chronic disease analogous to hypertension or diabetes, characterizing management as an endless journey of cyclical outpatient follow‐ups, medication adjustments, and hematologic monitoring.

#### 4.3.1 Symptom Burden

Fatigue was frequently reported as a predominant constitutional symptom. Unexplained dizziness or vertigo was also common, particularly in PV and ET patients.

Normal people get tired after exercise, but mine's different—just climbing stairs wipes me out, and no amount of resting helps. When I get breathless walking to the mailbox, people assume I'm just out of shape or lazy… they have no idea how awful this really feels.

(N14, ET)

On bad days, the dizziness hits me multiple times—everything goes blurry, and sometimes I even lose vision completely for a few scary minutes before it comes back.

(N19, PV)

#### 4.3.2 Side Effects

MPN patients received pharmacologic treatments including oral agents (e.g., hydroxyurea, ruxolitinib) or subcutaneous interferon‐α (IFNα). Although targeting the underlying hematologic disorder, these therapies were associated with adverse effects, including gastrointestinal disturbances and pain syndromes.

I was on hydroxyurea for a while, but then my legs started ballooning up and every joint hurt so bad I could barely walk. Ended up having to quit the meds.

(N23, PMF)

Patients with PV or ET receiving long‐term IFNα therapy frequently reported injection site pain and associated emotional distress.

I've been receiving IFNα injections for the past 8 years. I experienced discomfort in my buttocks, pain in my shoulders, and a general aching throughout my body [with a frowning and painful look].

(N18, PV)

#### 4.3.3 Body Image Change

Splenomegaly was markedly more prevalent among participants with PMF. The enlarged spleen impaired daily function and altered body image, evoking feelings of inferiority, shame, and helplessness.

I felt full after eating just a tiny amount of food, and when I go out for a walk, the neighbours often mistook me for being pregnant.

(N07, PMF)

Patients with PV frequently present with facial erythema and pruritus, precipitating body‐image disturbances and associated distress.

(The patient removed his mask to show) You see, my eyes and cheeks are red, and this (pointing to the cheek) looks like senile plaques. My face was once clear, but following the initiation of treatment, the plaques have begun to appear.

(N15, PV)

### 4.4 Theme 2: The Sword of Damocles

Despite symptomatic relief from chemotherapeutic and targeted agents, MPN remains a chronic progressive illness with heterogeneous prognosis, substantial financial toxicity, and limited disease awareness. Patients consistently reported “Sword of Damocles” anxiety, especially during disease progression.

#### 4.4.1 Uncertainty of the Future

Over half of the participants acknowledged accepting disease progression uncertainty and limited therapeutic efficacy despite regular treatment and follow‐up.

At first, I was very emotional and temporarily unable to accept it. I might have thought this illness could suddenly disappear. Now my head gets it, but every hospital visit… seeing nothing's changed… (sigh) Who knows what's coming next.

(N17, ET)

#### 4.4.2 Fear of Disease Progression

Several younger participants expressed concerns that disease exacerbation could negatively impact their families, influenced by their familial and social roles.

(voice shaking) I'm only in my thirties… got diagnosed right after the wedding. Now I'm terrified it'll turn into leukemia, and it kills me to put my wife and parents through this.

(N05, PV)

My physical condition was not very good when I was a teenager. Now, I haven't yet gotten married or started a career. I've just participated in the work for a few years to ease my parents' worries. But I'm always waiting for the other shoe to drop with my health.

(N13, PV)

#### 4.4.3 Cognitive Avoidance

Some patients were still reluctant to face the disease since they had been diagnosed. They avoided “understanding too much” to protect their psychological well‐being.

Right now I'm stable, and I'd rather stay in the dark about details. Perhaps I have 10 good years left if I don't ask questions, but it could be just 2 months if I hear the truth.

(N09, PV)

### 4.5 Theme 3: Role Conflict

Participants' strong motivation to resume normalcy, stemming from their multifactorial social roles, created significant role conflict between daily responsibilities and treatment adherence during prolonged recovery periods.

#### 4.5.1 Restricted Family Role

Almost all participants mentioned that disease impacts familial stability and interpersonal dynamics, with variable effects across households.

Well, I'm worrying more about my father. I thought I could take care of him, but I'm also worried that my child and husband will be burdened once my disease gets worse.

(N01, PMF)

I can still handle things on my own right now, so I'll do my part to not be a burden on my family. What matters is that as the child grows, she'll naturally look up MPN details herself. So I'll do everything I can to keep our family life running smoothly.

(N17, ET)

#### 4.5.2 Resistance to Returning to Work

Nearly half of patients reported occupational stress upon returning to work, primarily due to reduced work capacity and treatment‐related functional limitations.

(sighs) I'm really struggling with being on medication for life. All the check‐ups and sick days are just too much to handle. At work, I have to carry pills every day but hide them from colleagues…It's just unsustainable long‐term.

(N20, PMF)

#### 4.5.3 Reluctant to Disclose the Condition

Approximately half of participants concealed their diagnosis to avoid perceived vulnerability and minimize social scrutiny.

Everyone thought I was perfectly healthy since I always came across as so positive. When I had hospital check‐ups, I'd just say I was going on a little getaway—no reason to bother them with the details, you know?

(N17, ET)

If I apply for jobs and they find out about my condition? No way I'd get hired. Once this private stuff comes out, people just label you.

(N20, PMF)

### 4.6 Theme 4: Systemic Barriers

#### 4.6.1 Lack of Professional Support

Some patients reported that common symptoms and psychological distress often lacked individualized management, as these were frequently dismissed as inherent disease features.

I often feel exhausted, but during clinic visits, the doctors can't really do much about it. For patients like me, we just have to learn to manage our energy and time wisely.

(N01, PMF)

#### 4.6.2 The Information Paradox

Some patients primarily sought disease‐related information through online searches, despite variable quality. The rapidly expanding volume of such information often exceeded patients' processing capacity, creating a pervasive dilemma.

My condition was first flagged during a routine check‐up. When I searched online, the information was too technical to understand. It wasn't until I started regular follow‐up visits at the clinic that I connected with other patients and finally joined an online support group.

(N16, ET)

Sometimes after my check‐ups, I try to look up my blood test results online to understand what they mean. However, there's just too much information out there, reading it all makes me even more anxious.

(N10, ET)

#### 4.6.3 Lack of Empowerment

Participants stated that there would be a greater need for individuals to pay attention to their management strategies, which could help them have a sense of control.

After tracking my fatigue patterns for months, I finally realized pacing myself with 20‐minute rest periods between tasks makes a difference. It's not a cure, but at least I feel less powerless when I can predict the crashes.

(N25, PMF)

My hematologist only checks my blood counts, but what helps me cope is the symptom diary the nurse suggested. Seeing the small improvements week by week keeps me from drowning in anxiety.

(N26, PMF)

## 5 Discussion

This study mapped health‐related distress and challenges among Chinese MPN patients in outpatient settings, identifying four key themes: living with the illness, the sword of Damocles, role conflict, and systemic barriers. Patients universally acknowledged the chronicity of MPN, highlighting the need for integrated long‐term support including treatment guidance, psychological care, and social services during routine follow‐up.

MPN patients in this study predominantly reported constitutional symptoms including fatigue, weight loss, night sweats, and splenomegaly, consistent with Tremblay and Mesa [18] findings. In addition, cross‐cultural validations, such as the Romanian MPN‐10 adaptation, further identify fatigue, inactivity, and concentration difficulties as particularly burdensome [21]. Notably, Cancer‐related fatigue manifested distinctively from normal tiredness, causing systemic discomfort and cognitive impairment [22]. Bradford's research reported that the self‐care strategies used by the MPN participants, such as “taking care of my body” and “spending and saving my energy” [12] were closely associated with fatigue severity and modulated by biopsychosocial factors. Furthermore, body image disturbances significantly contributed to distress in MPN patients. Consistent with observations in breast and colorectal cancer [23, 24], these alterations impaired self‐esteem and reduced body satisfaction, especially among young and middle‐aged adults.

Some participants also reported treatment‐related side effects from injections or oral medications. Hydroxyurea and IFNα are classical cytoreductive agents used as first‐line and second‐line treatments for patients with PV and ET to reduce thrombotic burden [25]. Consistent with the review [26], IFNα requires multiple injections per week or month and is often poorly tolerated because of non‐hematological adverse effects such as pain, headache, fatigue, flu‐like symptoms, and depression. Moreover, approximately 20% of patients discontinued IFNα due to side effects, with the majority of discontinuations occurring in the first year of treatment [27]. These findings underscore the necessity for hematology specialist teams to provide tailored injection regimens and patient education.

This study revealed significant patient psychological distress at diagnosis, including depression and loss of life meaning. Patients acknowledged medical efficacy for physical symptoms, whereas psychological comorbidities (anxiety: 21%; depression: 12%; both: 8%) remained predominantly self‐managed [28]. Additionally, fear of progression and uncertainty were primary triggers of negative emotions, frequently leading to maladaptive coping. Importantly, psychological distress persisted despite physical symptom improvement, as fatigue and functional decline exacerbated social withdrawal and reduced quality of life. Surapaneni and Scherber [29] recommended non‐pharmacological interventions such as yoga, mindfulness, meditation, and nutritional supplements to alleviate physical and psychological burdens, thereby improving long‐term quality of life in MPN patients.

MPN patients frequently experienced role conflicts involving restricted family functioning, occupational limitations, and illness non‐disclosure. These conflicts arise from competing role demands exceeding coping capacity, as explained by role theory [30]. Consistent with prior research, symptom burden often impedes work resumption, with studies indicating 24.8% of patients require medical leave and 62.6% of those fail to return [31]. Furthermore, fatigue significantly impairs daily functioning across multiple domains [12]. Consequently, early family‐focused interventions should promote shared disease understanding and stress management to enhance resilience and relational dynamics.

This study identified critical systemic barriers in MPN care: insufficient professional support, an information paradox, and untapped patient empowerment. Patients reported that common symptoms like fatigue were often dismissed, leading to self‐developed coping strategies, which align with reports of under‐addressed quality‐of‐life issues in MPN [32]. While clinicians may provide credible resources, information overload frequently outweighs scarcity, leading to decision fatigue and potentially impaired adherence [33]. Although patients demonstrated adaptive self‐management (e.g., fatigue diaries), these strategies remained largely self‐taught, reflecting broader gaps in supportive care [34]. These findings underscore the need for improved clinician training in invisible symptoms, standardized patient education, and integration of self‐management tools into routine care to enhance holistic management without compromising efficacy.

### 5.1 Limitations

To our knowledge, this is the first study to explore health distress and challenges among Chinese MPN patients in outpatient follow‐up settings. However, the relatively small subtype‐specific sample sizes may limit comprehensive analyses. Additionally, the limited representation of long‐term disease duration (only four participants > 10 years) may not fully capture the experiences across this heterogeneous disorder. Future studies should include larger cohorts with stratified disease durations and subtype‐specific analyses to better understand patient experiences.

## 6 Conclusions

The health distress and challenges experienced by patients with MPN were explained by themes of living with the illness, the sword of Damocles, role conflict, and systemic barriers. The chronic trajectory of MPN necessitates long‐term outpatient care, during which patients increasingly prioritize symptom control, self‐management, and functional preservation. Most patients ultimately seek social reintegration rather than perpetual illness‐focused adjustment, reflecting a fundamental desire for normalcy. This study provides a foundation for developing interventions to empower MPN patients during stable disease phases.

## Author Contributions

**Yating Liu:** formal analysis (equal); writing – original draft (lead). **Jinying Zhao:** formal analysis (equal); writing – review and editing (supporting). **Qianqian Zhang:** patient recruitment (lead); methodology (supporting). **Qingyan Gao:** methodology (supporting); patient recruitment (equal). **Yayun Cao:** formal analysis (equal); writing – review and editing (supporting). **Jia He:** conceptualization (equal); writing – review (supporting). **Lihua Han:** arrangement of outpatient clinic (supporting); conceptualization (equal). **Yandi Wang:** arrangement of outpatient clinic (lead); conceptualization (equal). **Qian Yang:** conceptualization (equal); supervision (supporting). **Wenjun Xie:** methodology (lead); project administration (lead); supervision (lead); writing – review and editing (lead).

## Conflicts of Interest

The authors declare no conflicts of interest.

## Data Availability

The data are not publicly available because of the potential for participant identification and the ethical requirements for data release. Reasonable requests for additional information can be submitted to the corresponding author.
